# Neuropeptide Y and its receptors in prostate cancer: associations with cancer invasiveness and perineural spread

**DOI:** 10.1007/s00432-022-04540-x

**Published:** 2022-12-30

**Authors:** Dawid Sigorski, Wojciech Wesołowski, Agnieszka Gruszecka, Jacek Gulczyński, Piotr Zieliński, Sara Misiukiewicz, Joanna Kitlińska, Ewa Iżycka-Świeszewska

**Affiliations:** 1 Department of Oncology, Collegium Medicum, University of Warmia and Mazury, 10-228 Olsztyn, Poland; 2 Department of Oncology and Immuno-Oncology, Warmian-Masurian Cancer Center of the Ministry of the Interior and Administration Hospital, 10-228 Olsztyn, Poland; 3 EL-PAT Pathology Co, 82-300 Elbląg, Poland; 4 Department of Radiology Informatics and Statistics, Medical University of Gdansk, 80-210 Gdansk, Poland; 5 Department of Pathology and Neuropathology, Medical University of Gdańsk, 80-210 Gdańsk, Poland; 6 Department of Pathomorphology, Copernicus Hospital, 80-803 Gdańsk, Poland; 7 Division of Tropical and Parasitic Diseases, University Center of Maritime and Tropical Medicine, 81-519 Gdynia, Poland; 8 Human Science Department, School of Nursing and Health Studies, Georgetown University Medical Center, Washington, DC USA; 9 Department of Biochemistry and Molecular and Cellular Biology, Georgetown University Medical Center, Washington, DC 20057 USA

**Keywords:** Neuropeptide Y, Perineural invasion, NPY receptors (Y1R, Y2R, Y5R), Prostate cancer, Bone metastasis, Tumour microenvironment

## Abstract

**Purpose:**

Neuropeptide Y (NPY) is a pleiotropic peptide, which is involved in many biological mechanisms important in regulation of cell growth and survival. The aim of this study was a comprehensive analysis of the NPY system in prostate pathology.

**Methods:**

The study was based on immunohistochemical analysis of NPY and its receptors, Y1R, Y2R and Y5R, in tissue samples from benign prostate (BP), primary prostate cancer (PCa) and PCa bone metastases. Tissue microarray (TMA) technique was employed, with analysis of multiple cores from each specimen. Intensity of the immunoreactivity and expression index (EI), as well as distribution of the immunostaining in neoplastic cells and stromal elements were evaluated. Perineural invasion (PNI) and extraprostatic extension (EPE) were areas of special interests. Moreover, a transwell migration assay on the LNCaP PCa cell line was used to assess the chemotactic properties of NPY.

**Results:**

Morphological analysis revealed homogeneous membrane and cytoplasmic pattern of NPY staining in cancer cells and its membrane localization with apical accentuation in BP glands. All elements of the NPY system were upregulated in pre-invasive prostate intraepithelial neoplasia, PCa and metastases. EI and staining intensity of NPY receptors were significantly higher in PCa then in BP with correlation between Y2R and Y5R. The strength of expression of the NPY system was further increased in the PNI and EPE areas. In bone metastases, Y1R and Y5R presented high expression scores.

**Conclusion:**

The results of our study suggest that the NPY system is involved in PCa, starting from early stages of its development to disseminated states of the disease, and participates in the invasion of PCa into the auto and paracrine matter.

## Introduction

Cancer neurobiology constitutes a new and fast developing discipline. The involvement of the nervous system in cancer development and progression has been identified as one of the hallmarks of cancer (Senga and Grose 2021). The role of neural signalling is complex and multifaceted, involving effects on carcinogenesis, cancer spread and interactions between the tumoural cells and the microenvironment (Boilly et al. 2017). Nerves modulate immune response, cellular proliferation, apoptosis, angiogenesis and stem cell niche, and affect cancer-induced pain by diverse pathomechanisms. Neoplastic stroma is a complex structure which provides structural and nutritional support for the proliferating cells by releasing multiple functional and signalling particles, including neurotransmitters and neuropeptides. Neural structures are present within different parts of neoplastic tumours and their periphery. The nerves in cancer include the pre-existent structures within the host organ and nerves, as well as new neuronal network formed by neoaxonogenesis from pre-existing nerves or transformed due to neuron reprogramming. Neuronal precursors may also migrate to tumoural mass from distant parts of the body (Ayala et al. 2008; Amit et al. 2016; Mauffrey et al. 2019; Mravec 2022). New forms of tumour–nerve cross talk is created in neoplasia via multiple mechanisms. One such form of interaction between cancer and nerves is perineural invasion, which not only constitutes a way of cancer spreading, but also creates a unique neural niche for neoplastic cells (Brown 2016; Chen et al. 2019). The important role in this process is played by axons, which secrete multiple trophic factors, and Schwann cells that support neurons and can also increase malignant cells’ invasiveness and motility and promote metastases (Deborde and Wong 2017; Shurin et al. 2020; Sun et al. 2022).

The role of the nervous system and neuronal signalling in pathobiology and the clinical course of prostate cancer (PCa) became a field of multidirectional research initiated by the series of studies by Ayala et al., who described the process of axonogenesis, neuronal–epithelial interactions and mechanisms of perineural invasion in PCa. Ayala’s group has also shown that cancer cells are capable of inducing neurite outgrowth (Ayala et al. 2001, 2004, 2006, 2008). Accordingly, PCa develops in elderly men, mainly in the peripheral, best-innervated zone of the organ, being frequently multifocal and very often exhibiting signs of perineural invasion (Zareba et al. 2017). The natural history of PCa and ways of its progression are difficult to predict due to heterogenous and variable nature of this disease, which may range from indolent to highly aggressive. Assessment of progression risk is one of the most challenging aspects in PCa. There are several well-established prognostic pathoclinical factors, which provide a foundation for therapeutic planning. Treatment of localized PCa depends on the risk group related to PSA level, histological grade group, tumour stage, imaging and the age of the patient. The main first-line therapeutic modalities for localized disease include prostatectomy, radiotherapy or wait-and-see strategy. In advanced stage and disseminated disease, androgen deprivation therapy is the backbone of treatment. The main challenges in PCa include personalization of treatment, bone health and castration-resistant phase of the disease (Mottet et al. 2020; Rebello et al. 2021).

The normal prostate gland is richly innervated with nonhomogeneous nerve distribution, decreasing from the base to the apex of the gland. Moreover, adrenergic fibres innervate mainly stromal elements, while cholinergic fibre endings involve glandular cells of prostatic acini (Park et al. 2013; Magnon et al. 2013). Interestingly, in the normal prostate glands, basal cells express more proneural genes than luminal cells, many of which are typically associated with neural development, neurogenesis and axonal guidance. In parallel, the luminal cells express many genes related to neural signal response and processing (Zhang et al. 2016). Additionally, scattered neuroendocrine cells are present in normal prostate acini (Butler and Huang 2021). Noradrenaline and acetylcholine are the primary neurotransmitters; however, the prostate is also supplied by a wide range of neuropeptides that are physiologically, important starting from embryogenesis (Jen and Dixon 1995).

Axonogenesis and neurogenesis have been discovered for the first time in PCa as being involved in the early and late stages of PCa development (Ayala et al. 2008; Magnon et al. 2013; Mauffrey et al. 2019). In PCa, the normal pattern of prostate innervation changes in the context of its new forms of interactions with cancer cells and altered distribution (Sejda et al. 2020; Sigorski et al. 2021). These changes in nerve pattern lead to the alterations of neurotransmitter concentrations and neurosignalling control, which are key paracrine modulators of tumoural cells and stroma (Mancino et al. 2011). During androgen deprivation therapy, some PCa undergo neuroendocrine transdifferentiation, an androgen receptor-independent mechanism of castration resistance (Crona and Whang 2017). Neuroendocrine differentiation is promoted by not only androgen depletion, but also fractionated ionizing radiation, cAMP, cytokines (IL-6) and noradrenaline. Adrenergic signalling in tumours promotes angiogenesis, metastases and neuroendocrine differentiation, while a β-adrenergic antagonist, propranolol, inhibits some of these processes (Deng et al. 2008; Zhao and Li 2019; Dwivedi et al. 2021). Neuroendocrine cells contain neurosecretory granules, which store several peptide hormones involved in paracrine regulation of prostate gland functions. While the exact role of neuropeptides in PCa is not well elucidated, several potential mechanisms have been identified. The calcitonin gene-related peptide, gastrin-releasing peptide, parathyroid hormone-related protein, vasoactive intestinal peptide and bombesin increase invasiveness of PCa cells (Hoosein et al. 1993; Nagakawa et al. 2001). In addition, bombesin stimulates PCa cell growth and prevents apoptosis induced by chemotherapy (Bologna et al. 1989; Salido et al. 2002). Similarly, neurotensin has a mitogenic effect in PC3 and LNCaP cell lines (Amorino et al. 2006).

Neuropeptide Y (NPY) is a common neurotransmitter in the central and peripheral nervous system, acting via a system of G-protein-coupled receptors—Y1R, Y2R and Y5R (Larhammar and Salaneck 2004; Lin et al. 2004). NPY is the most abundant peptide in the sympathetic nerves and is co-released with noradrenaline. This peptide regulates many physiological processes, including hunger, behavioral reactions, energy homeostasis, blood pressure, bone metabolism and turnover (Pedrazzini et al. 2003; Gehlert 2004). NPY is synthesized in many neuronal populations, sympathetic and sensory nerves, adrenal medulla, as well as in endothelial cells, platelets and several epithelial cell types (Hirsch and Zukowska 2012). The cellular and tissue NPY expression is under the control of a variety of factors, such as neurotrophins, NGF and BDNF, which are the key regulators of development, differentiation and regeneration of nerves (Czarnecka et al. 2015). Expression of NPY was shown in precursors of Schwann cells, which play a role in nerve fasciculation, regeneration and maturation (Ubink and Hökfelt 2000).

NPY protects neurons from injury, interacts with BDNF and regulates nutritional support (Zhang et al. 2021). NPY-dependent modulation of the proliferative potential was shown in cells with neuronal origin such as hippocampus, retina, neuronal precursors and injured glial cells (Álvaro et al. 2008; Decressac et al. 2011; Geloso et al. 2015). Moreover, this peptide promotes proliferation of olfactory epithelium and bone marrow-derived mesenchymal stem cells (Negroni et al. 2012; Wu et al. 2017). The cellular effects of NPY are mediated by several different intracellular signalling pathways, including adenylyl cyclase inhibition and a p44/42 mitogen-activated protein kinase (MAPK) stimulation (Ruscica et al. 2006).

The dysregulation of the NPY system is associated with various diseases, including diabetes, obesity, retinopathy, inflammatory conditions, neurodegenerative and neuroimmune disorders, and tumours. Growing evidences from pediatric tumours, neuroblastoma and Ewing sarcoma, as well as adulthood malignancies, such as breast, prostate and GI cancers, show its diverse and tumour type-dependent effects on neoplastic phenotype and tumour microenvironment. The NPY system regulates proliferation, differentiation, apoptosis and migration of neoplastic cells, as well as angiogenesis (Pedrazzini et al. 2003; Koulu et al. 2004; Ruscica et al. 2005; Li et al. 2011; Santos-Carvalho et al. 2013; Tilan et al. 2013; Zhang et al. 2014; Geloso et al. 2015; Hong et al. 2015; Abualsaud et al. 2021). Studies on Ewing sarcoma demonstrated the association between high systemic NPY levels and bone metastasis and provided direct evidence for the role of the NPY system in this process (Tilan et al. 2015; Hong et al. 2015; Lu et al. 2022). In line with these findings, several studies on NPY expression and function in PCa on human cancer tissue, cell lines and in animal models suggested its role in regulation of tumour cell proliferation, resistance to chemotherapy and metabolic adaptations (Alshalalfa et al. 2019; Ding et al. 2021; Sigorski et al. 2021).

NPY expression undergoes age-dependent modifications. Such changes were observed in animal brain, where they affect neuronal functions and modulate adrenergic signalling (Higuchi et al. 1991), as well as in other organs, such as liver (Dietrich et al. 2020). The presence of NPY in prostatic capsule is detectable starting from week 13 of embryogenesis, in smooth muscle bundles starting from week 17 and in acini of prostate starting at week 23 (Jen and Dixon 1995). NPY-containing prostatic nerves are probably the most abundant between 10 and 20 years of age, before the androgen axis is established (Ding et al. 2021). The number of NPY-positive nerve fibres is higher in the peripheral area of the prostate than in the anterior fibromuscular stroma (Iwata et al. 2001). Studies on periprostatic ganglions showed an increase in their number in peripubertal rats, as compared to prepubertal animals (Pozuelo et al. 2010). Several studies suggest that the NPY axis is involved in PCa biology, revealing different associations with genetic alterations, histology, tumour biochemical recurrence and patients’ outcome. In the Cancer Genome Atlas (TCGA) PAN-Cancer cohort, PCa exhibits the highest NPY expression among all examined cancers (Alshalalfa et al. 2019). This high NPY levels may result from both the endogenous peptide expression in tumour cells and its presence in neuronal structures. Recent studies revealed an important role of such neuronal NPY, which regulates the tumoural metabolism, apoptosis, motility and therapy resistance via activation of the Y1R (Ding et al. 2021). Consequently, the number of NPY-positive nerves was predictive for PCa-specific death, biochemical recurrence and radiation therapy resistance (Ding et al. 2021).

However, there is a lack of comprehensive studies on tissue expression of NPY and its receptors Y1R, Y2R and Y5R in PCa, as well as functions of the peptide in this malignancy. Activated NPY receptors regulate the proliferative potential of PCa cells via various mechanisms. NPY inhibits proliferation of LNCaP, DU145 cell lines, while it acts as a mitogenic factor for androgen-independent PC3 cells (Magni and Motta 2001; Ruscica et al. 2006).

Osteoblastic lesions are a typical form of prostate cancer metastases and may lead to skeletal-related events, worsening quality of life and decreasing survival in PCa patients. The NPY system controls bone metabolism, formation and resorption, mobilizes hematopoietic stem cells and stimulates angiogenesis. Altogether, these direct effects of NPY on bone homeostasis may contribute to bone metastasis, as previously postulated (Chen and Zhang 2022).

We postulate that the NPY system expression differs between benign prostate and cancer tissue and relates to some pathoclinical tumour characteristics. In the tumour microenvironment, NPY represents a common mediator of various neuronal and non-neuronal effects, since it is synthesized by tumoural cells and nerves. In this study, the topographic and quantitative aspects of the NPY system expression were assessed in tissues from benign prostate, PCa and bone metastases in the context of selected morphological features and prognostic traits. In addition, we studied the chemotactic effects of NPY on PCa cells. The research on the structure and function of tumour neuroenvironment is crucial for better understanding of the PCa biology and developing new diagnostic and treatment options in the future.

## Materials and methods

### Study group

The research was approved by the Bioethical Committee of the Medical University of Gdansk no. NKBBN/448/2015. The study material consisted of PCa tissue sections taken from radical prostatectomy specimens and spinal metastasectomies of PCa from archival resources covering the period 2012–2014. From 85 pre-selected PCa cases, 51 primary tumours and 11 bone metastases were enrolled in the study (Table 1). The database was constructed and anonymised according to the institutional regulations. Two cases came from the autopsies of the patients with untreated PCa with bone metastases. The selection criteria of the samples included: quantity and representativity of neoplastic tissue, and various tumour stages. The available pathoclinical data included the age of the patients, the Gleason score, grade group, pTNM, presence of perineural invasion (PNI) and extraprostatic extension (EPE). Tumour staging and histologic classification were based on the American Joint Committee on Cancer (AJCC) tumour/node/metastasis (TNM) classification, 8th edition. The examined group consisted of tumours in stages pT2a (*n* = 5), pT2b (*n* = 1), pT2c (*n* = 28), pT3a (*n* = 12), pT3b (*n* = 4) and pT4 (*n* = 1) (Table 1). In three cases, lymph node metastases were present. The study was performed on routinely processed FFPE tissue sections with tissue microarray (TMA) technique with analysis of multiple cores from each specimen. Moreover, the whole tissue specimens were assessed for the detailed expression topography evaluation, the presence of the extracapsular extension or perineural invasion areas, and this information was used to guide tissue collection for further analyses.

**Table 1:** Clinicopathological characteristics of the study group

Group	BP and benign cancer periphery	PIN	PCa	BM
*n* =	6 + 20 (12 TMA cores + 20)	30	51 (167 TMA cores)	11 (18 TMA cores)
Patients’ age (median, range)	71 (61–78)	64 (45–75)	61 (45–75)	61 (51–70)
pT	–	–	*n* (%)	–
2a			5 (9.80)	
2b			1 (1.96)	
2c			29 (56.86)	
3a			12 (23.53)	
3b			4 (7.84)	
EPE^a^	–	–	*n* (%)	–
Positive			13 (25.49)	
Negative			38 (74.51)	
PNI ^b^	–	–	*n* (%)	–
Positive			27 (52.94)	
Negative			24 (47.06)	
Grade group	–	–	*n* (%)	–
1			15 (29.41)	
2			11 (21.57)	
3			8 (15.69)	
4			10 (19.61)	
5			7 (13.73)	
ERG	–	–	*n* (%)	*n* (%)
Positive			23 (45)	2 (22)
Negative			28 (55)	9 (78)
Ki-67 (median, range; %)	(0–1)	–	4 (1–25)	2 (1–10)

*BM* bone metastases, *BP* benign prostate, *EPE* extraprostatic extension, *PCa* prostate cancer, *PIN* prostate intraepithelial neoplasia, *PNI* perineural invasion

^a^Material for EPE analysis was avalaible in 13 cases from 16 cases with pT3 tumours

^b^In the pre-selected series of PCa, PNI was seen in 82% of the cases, but in the study group PNI was found in 52.94% of the cases

Tissue samples were assembled in TMAs with cores of 3–5 mm diameter placed in recipient blocks (UNITMA® Manual Tissue Microarrayer, Quick Ray /UT06/; UNITMA® Premade Recipient Blocks). Finally, 167 cores from PCa (each cancer case was represented by 2–4 cores) and 18 samples of bone metastases were examined. The control non-neoplastic prostate group consisted of 6 cases of the prostatectomies due to benign hyperplasia and 20 PCa specimens containing areas without neoplastic infiltration. Moreover, 30 cases with prostate intraepithelial neoplasia (PIN) coexisting with PCa infiltration were the second examined group. In seven cases of PCa and three cases of BP, a topographic assessment on complete tissue sections was performed in addition to TMAs. Non-neoplastic prostate tissue was included in 32 cores. All TMAs and full tissue sections were cut serially on ten slides, where the first and tenth were stained with H&E, while the rest of the sections were used for IHC.

### Immunohistochemistry

A ready-to-use system was used to perform the immunohistochemical reactions: DAKO EnVision™ FLEX+, Mouse, Low pH (Link), catalogue number K8002, and PT-Link system (Dako). The following antibodies were used: anti-NPY (ab30914, polyclonal, rabbit, 1:2000, Abcam), anti-NPY1R (ab183108, polyclonal, rabbit, 1:25, Abcam), anti-NPY2R (PA5-41576, polyclonal, rabbit, 1:400, Thermo Fisher), anti-NPY5R (ab32886, polyclonal, goat, 1:100, Abcam), anti-ERG (ab136152, monoclonal, mouse, 1:20, Abcam), anti-Ki67 (M7240, monoclonal, mouse, DAKO, Ready-to-Use). Antibody Diluent with Background Reducing Components (DAKO, Code S3022) was used. The procedures were performed according to a standard immunostaining procedure, with primary antibody incubation for 30 min at room temperature. Positive and negative controls recommended by the manufacturers were used.

### Criteria of immunostaining assessment

The immunoreactivity intensity of NPY and its receptors—Y1R, Y2R and Y5R—was assessed in semiquantitative scale: 0 (negative, very low), 1+, 2+, 3+ (positive, with increasing intensity/quantity, respectively) twice by two observers (WW, EIS) with Olympus BX50 microscope. The tissue expression was evaluated separately in epithelial cells and stromal elements such as myofibroblast or fibroblasts, blood vessels and inflammatory cells. The subcellular localization—cytoplasmic, membranous and nuclear was specified. Moreover, tissue distribution—section topography analysis of the staining in cancer infiltrate was separated into the central zone, front of invasion, perineural invasion (PNI) and extraprostatic extension (EPE). The structures for the internal control of the NPY system expression included autonomic ganglia, nerves/axons and endothelial cells. Because of the heterogeneity of the staining intensity, the expression was assessed with expression index (EI) established according to the method by Pirker et al. with own modification (Pirker et al. 2012). The scale 0–300 points was used, based on the formula: EI: 1 × [% of cells staining weakly (0–1+)] + 2 × [% of cells staining moderately (2+)] + 3×[% of cells staining strongly(3+)]. Finally, EI was calculated according to the scale: EI 0 (0–49), EI 1 (50–99), EI 2 (100–199) and EI 3 (200–300). EI was assessed in subgroups, PCa, BP and PIN, and separately in EPE and PNI areas of special interest, where additional EI 4 was introduced for NPY immunostaining to reflect a stronger expression than that observed in cancer infiltration. For further analyses, EI was also divided into low and high categories (EI < 2 and EI ≥ 2, respectively). Based on the presence of nuclear ERG staining, the cases were divided into categories of ERG negative (no immunoreactivity) and ERG positive. Proliferative index Ki67 was assessed as percentage (%) of immunostained nuclei in hot spot areas under 200 × magnification.

### Cell culture

LNCaP PCa cells were obtained from the Georgetown University Tissue Culture and Biobanking Shared Resource and cultured in RPMI media supplemented with 10% foetal bovine serum (FBS), penicillin (200 units/mL), streptomycin (200 µg/mL) and fungizone (1 µg/mL).

### Chemotactic assay

The BD FluoroBlok™ 96-well transwell plate system (BD Biosciences, San Jose, CA) was used to evaluate cell migration. LNCaP cells were suspended in RPMI media supplemented with 5% FBS and seeded in the upper chambers at a density of 1 × 10^4^ cells per well. The lower chambers contained the same media supplemented with NPY at concentrations ranging from 10^−10^ to 10^−7^ M. The transwell migration plate was then incubated for 22 h at 37 °C, in 5% CO_2_, followed by staining with calcein AM at a concentration of 4ug/ml in Hank’s balanced salt solution (HBSS, Thermo Fisher Scientific). The fluorescence was measured from the bottom of the migration plates using EnSpire Multimode Plate Reader (Perkin Elmer, Waltham, MA).

### Real-time RT-PCR

RNA from LNCaP cells was isolated using High Pure RNA Isolation Kit (Roche Applied Science, Indianapollis, IN). cDNA was synthesized using iScript cDNA Synthesis Kit and amplified using ICycler iQ Detection System (Bio-Rad Laboratories, Hercules, CA), TaqMan Universal PCR Master Mix and pre-designed primers and fluorescein-labeled probes (Applied Biosystems, Foster City, CA). The results were calculated by the comparative CT method using β-actin as a reference gene.

### Statistical methods

Statistical analysis was performed with Statistica 13 Software and GraphPad Prism 6. All variables used in the experiment were measured on an ordinal scale, and their qualitative nature dictated the type of statistical analysis used to develop the results. The significance of the correlation coefficients V-Cramer was assessed using the non-parametric Chi-square test. The differences between the reaction strength of individual system components in the corresponding areas of PCa, PNI, EPE and BP derived from the same tumour were assessed using the Wilcoxon-matched pairs test. The Mann–Wittney test was used to test the differences in NPY system expression between tumours grouped based on their stage, ERG status and the presence of PNI and EPE. The value of 0.05 was adopted as the significance level of α. The differences in the chemotaxis experiment were calculated with the one-way repeated measure ANOVA, followed by Dunnett's multiple comparisons test.

## Results

### Morphological analysis of the NPY system expression in benign prostate and PCa

#### NPY

**Fig. 1:**
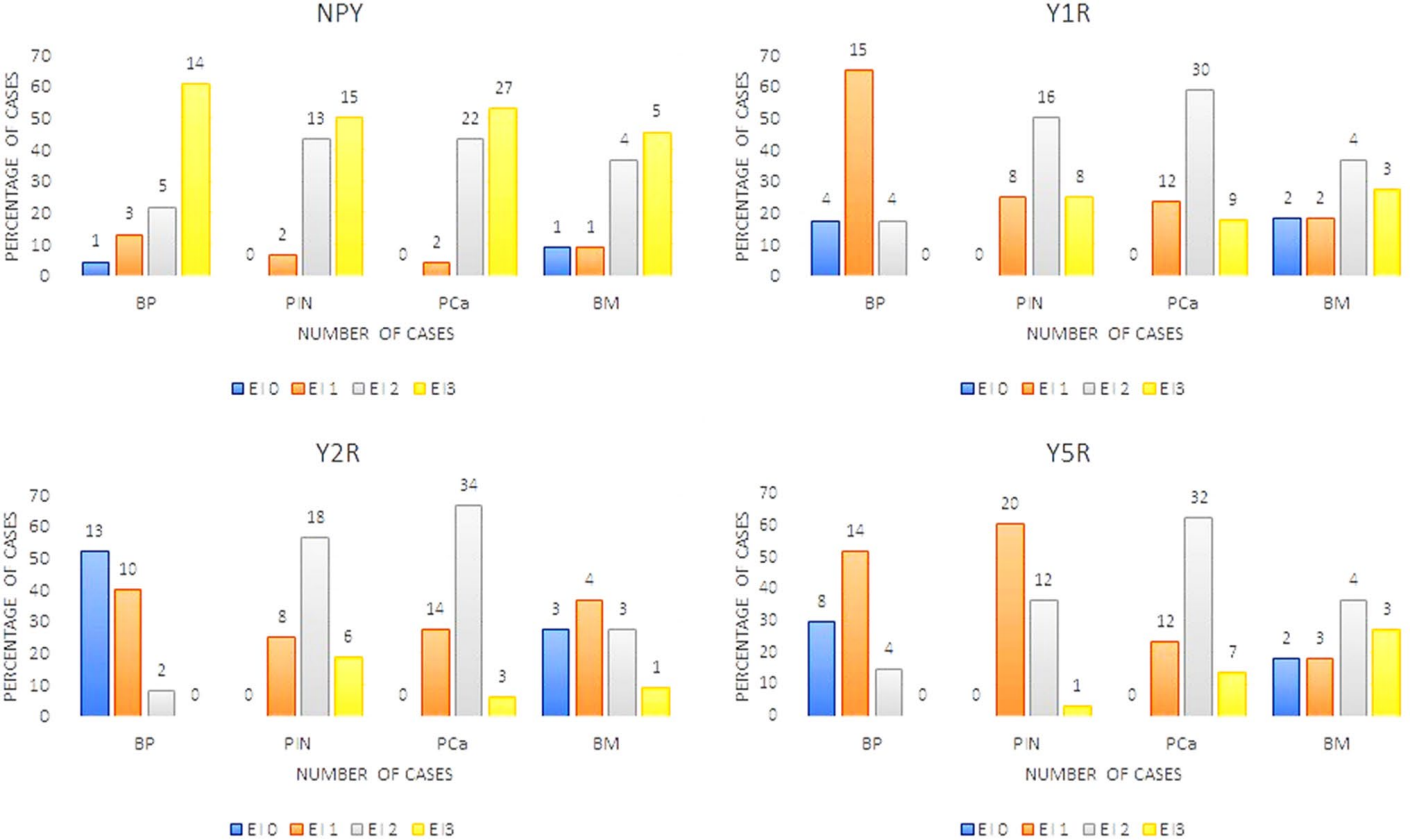
The NPY system expression scores in the study group. Abbreviations: *BP* benign prostate, *EI* expression index, *PIN* prostate intraepithelial neoplasia, *PCa* prostate cancer, *BM* bone metastases

**Fig. 2:**
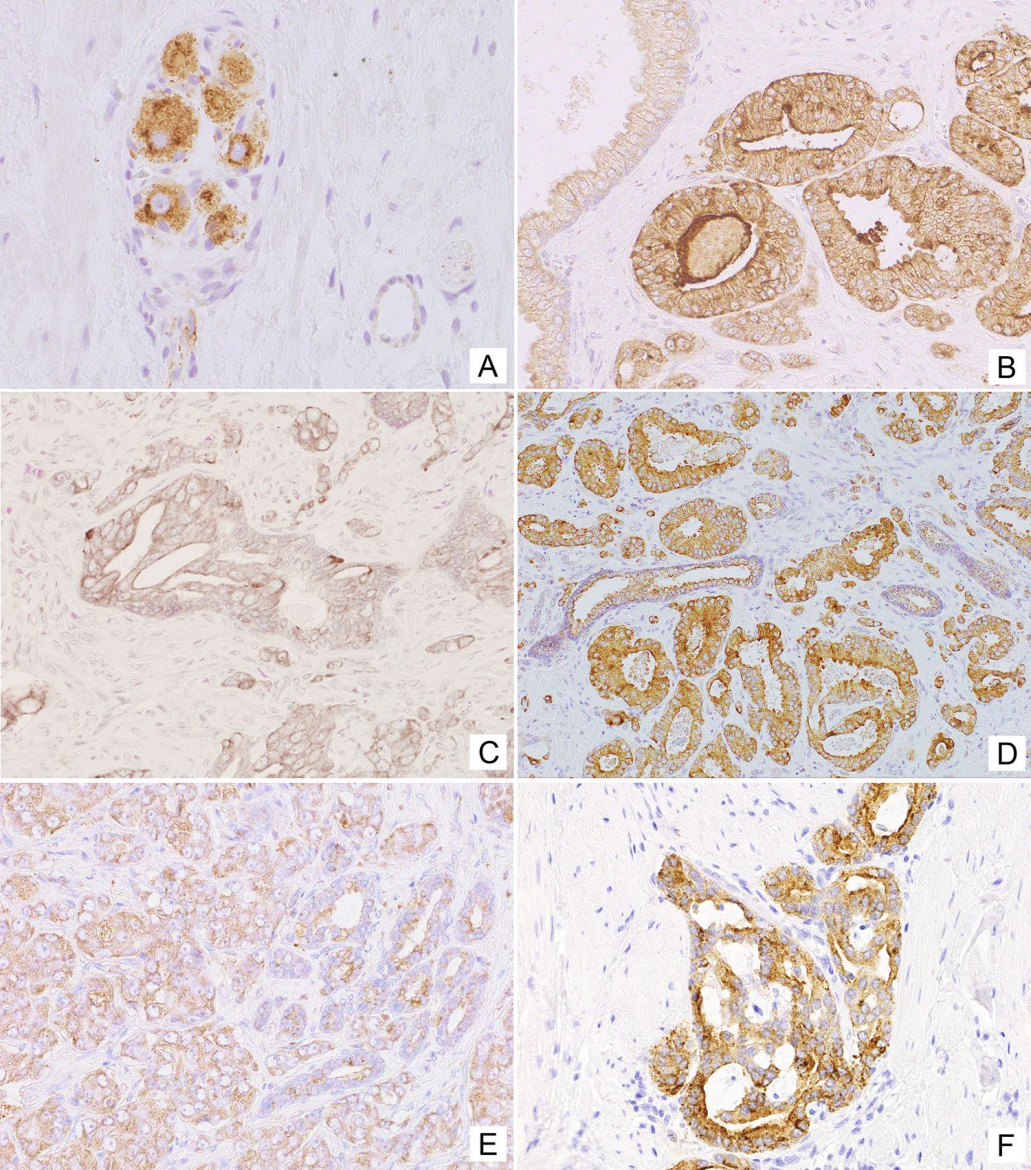
Tissue expression of NPY. **A** NPY-positive ganglion cells and axons (400×). **B** Membranous staining with apical accentuation within the normal prostate glands (400×). **C** Prostate intraepithelial neoplasia (PIN) with stronger in part cytoplasmic NPY expression (400×). **D** Strong, mainly cytoplasmic NPY immunoreactivity in malignant glands and luminal excretion (200×). **E** Low immunostaining in PCa (EI 1) (200×). **F** Strong NPY reactivity in bone metastasis (200×)

In line with known NPY expression in neuronal cells, positive immunostaining for NPY was observed in ganglion cells and some nerves within normal and cancerous prostate tissue (Fig. 2A). Moreover, non-neoplastic prostate showed membranous expression of NPY, mainly within the glandular luminal cells with apical accentuation, sometimes with a coexisting low cytoplasmic staining, and within the glandular secretion (Figs. 1, 2B). NPY EI was high (≥ 2) in 83% of cases (Table 2). Neuroendocrine cells showed intensive positivity with membranous and cytoplasmic pattern, while the basal cells were usually negative (Fig. 2B). NPY staining was more pronounced in atrophic glands. Focal reactivity in stromal component, such as lymphocytes and endothelial cells was visible in some cases.

**Table 2:** Expression index (EI) of NPY and its receptors in prostate tissue

	NPY EI (%)		Y1R EI (%)		Y2R EI (%)		Y5R EI (%)	
	L	H	L	H	L	H	L	H
BP^a^	17.39	82.65	82.65	17.39	92	8	81.48	18.52
PIN^a^	6.66	93.34	25	75	25	75	60.61	39.39
PCa^a^	3.92	96.08	23.53	76.47	27.45	72.55	23.53	76.47
EPE^b^	0	100	0	100	7.69	92.31	0	100
PNI^b^	0	100	8	92	3.7	96.3	0	100
BM^a^	18.8	81.92	36.36	63.64	63.63	36.37	36.36	63.64

*BM* bone metastases, *BP* benign prostate, *EPE* extraprostatic extension, *H *high, *L *low, *PCa* prostate cancer, *PIN* prostate intraepithelial neoplasia, *PNI* perineural invasion

^a^Expression index: low 0–1, high 2–3

^b^Expression index for NPY: low 0–1, high 2–4

High NPY expression was also observed in pre-invasive PIN lesions (Fig. 2C). Similarly, PCa glandular structures in the primary tumours and bone metastases revealed cytoplasmic and membranous diffuse or granular staining, heterogeneously distributed within the tumour infiltration with predominant EI 2 or EI 3 (high in 93% of cases; Figs. 1, 2D, E; Table 2). The staining intensity in neoplastic cells was often equal or higher than in ganglionic cells.

#### Y1R

**Fig. 3:**
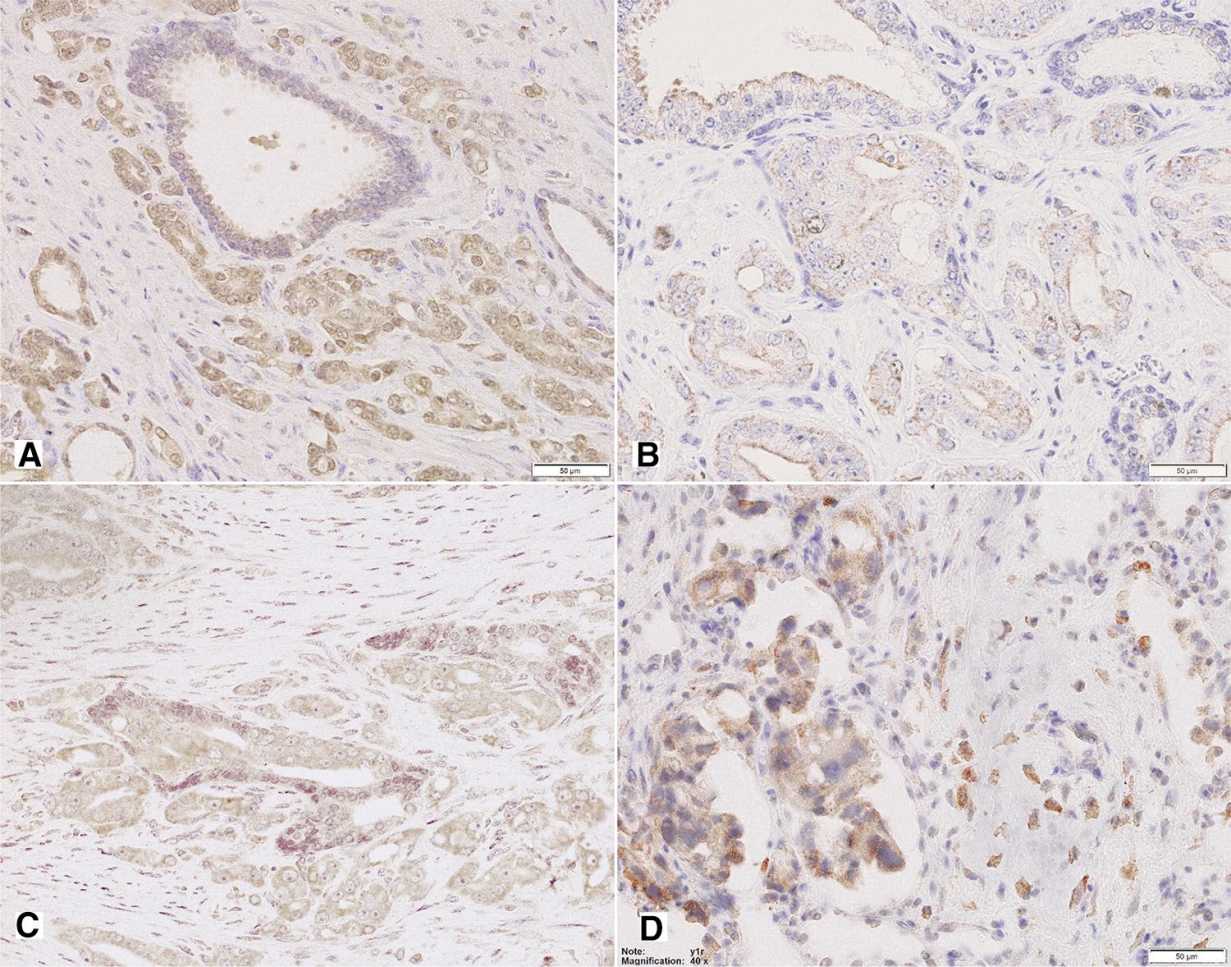
Tissue expression of Y1R. **A** Low membranous Y1R immunoreactivity within pre-existent prostate glands. **B** Increased cytoplasmic and membranous Y1R staining in cancer and PIN (400×). **C** High, mainly cytoplasmic Y1R staining within PIN and invasive cancer and positive myofibroblasts (400×). **D** Strong Y1R reactivity within cancer bone metastasis and osteoblasts (400×)

Non-neoplastic prostate showed low Y1R cytoplasmic and membranous expression in glandular luminal cells (82%, including 17% of cases with EI 0) and basal layer negativity (Figs. 1, 3A; Table 2). Y1R low staining was also identified in prostate stromal elements—in the muscular layer of blood vessels, myofibroblasts and some mononuclear inflammatory cells.

In PIN, PCa and bone metastases, Y1R immunoreactivity was cytoplasmic and membranous, homogenous and stronger than in BP structures, with the most frequent expression level of EI 2 (50, 58 and 36,4%, respectively; Figs. 1, 3B–D; Table 2). In 2 out of 11 bone metastases, no expression was found. Strong Y1R expression characterized bone osteoblasts (Fig. 3D).

#### Y2R

**Fig. 4:**
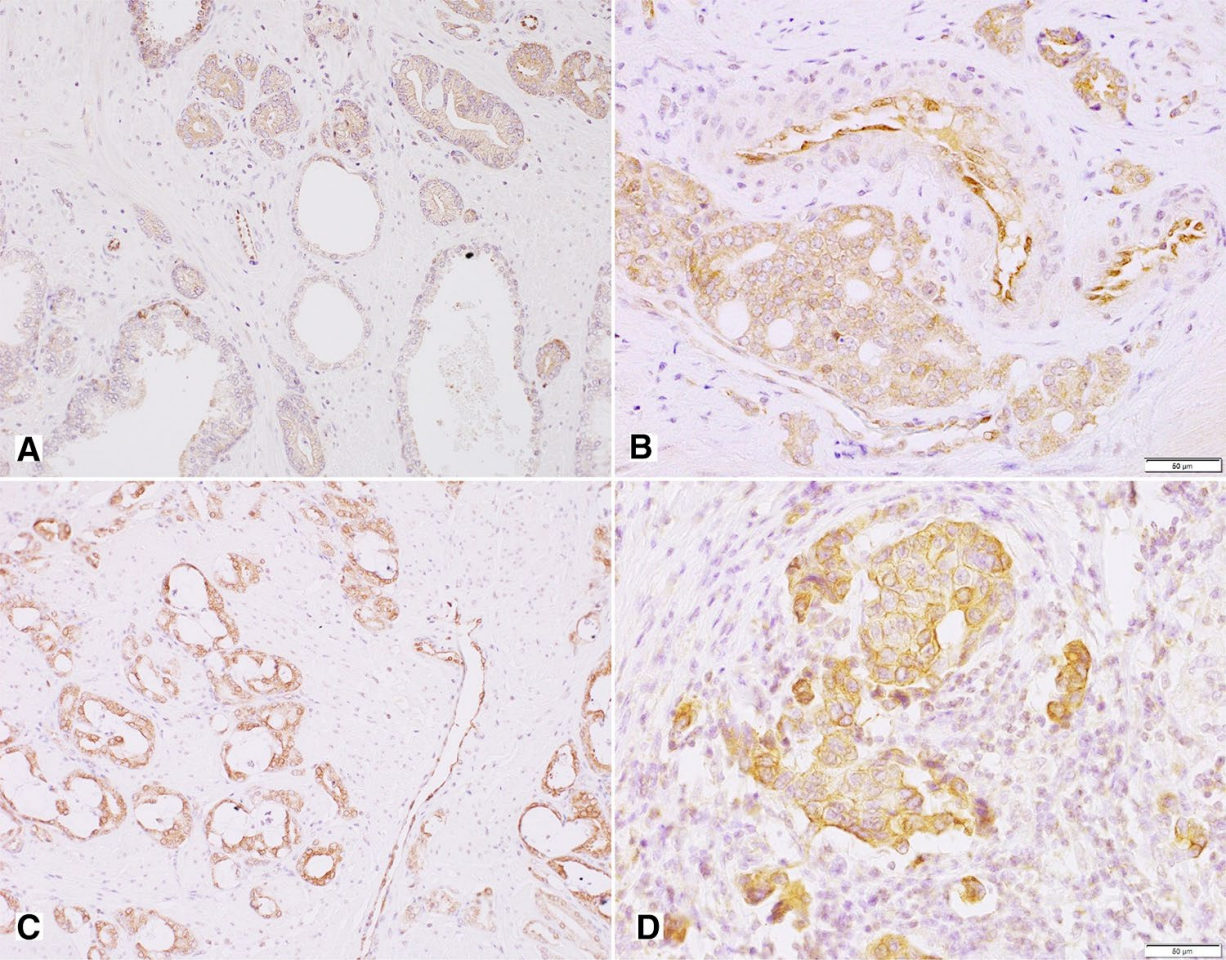
Tissue expression of Y2R. **A** Low Y2R expression within the benign prostate (200×). **B, C** High, mainly cytoplasmic reaction within cancer and endothelial cells (B 400×, C 200×). **D** Bone metastasis with high membranous and cytoplasmic reaction and low positivity in lymphocytes (400×)

In non-neoplastic prostate, cytoplasmic and membranous Y2R expression was present within glandular luminal cells, showing low EI in 90% of cases, encompassing EI 0 in half of them (Figs. 1, 4A; Table 2). Focally also, basal cells were immunoreactive. Atrophic glands showed higher Y2R reactivity. In the stroma, expression was found in endothelial cells (Fig. 4C).

In PCa, neoplastic cells revealed cytoplasmic and membranous Y2R staining, with heterogenous intensity, with prevalent EI 2 (66.6%) (Figs. 1, 4B, C; Table 2). In PIN, the level of expression was similar to that in invasive cancer (Fig. 4A). In cancer stroma, Y2R staining was detected in endothelial cells. In bone metastases, immunoreactivity was diverse with mostly low EI (63.4%) (Figs. 1, 4D; Table 2).

#### Y5R

Non-neoplastic prostate showed low cytoplasmic Y5R expression with membranous localization in glandular luminal cells, including 30% of cases with EI 0 (Fig. 1; Table 2). In the stroma, the expression was found in the endothelial cells.

**Fig. 5:**
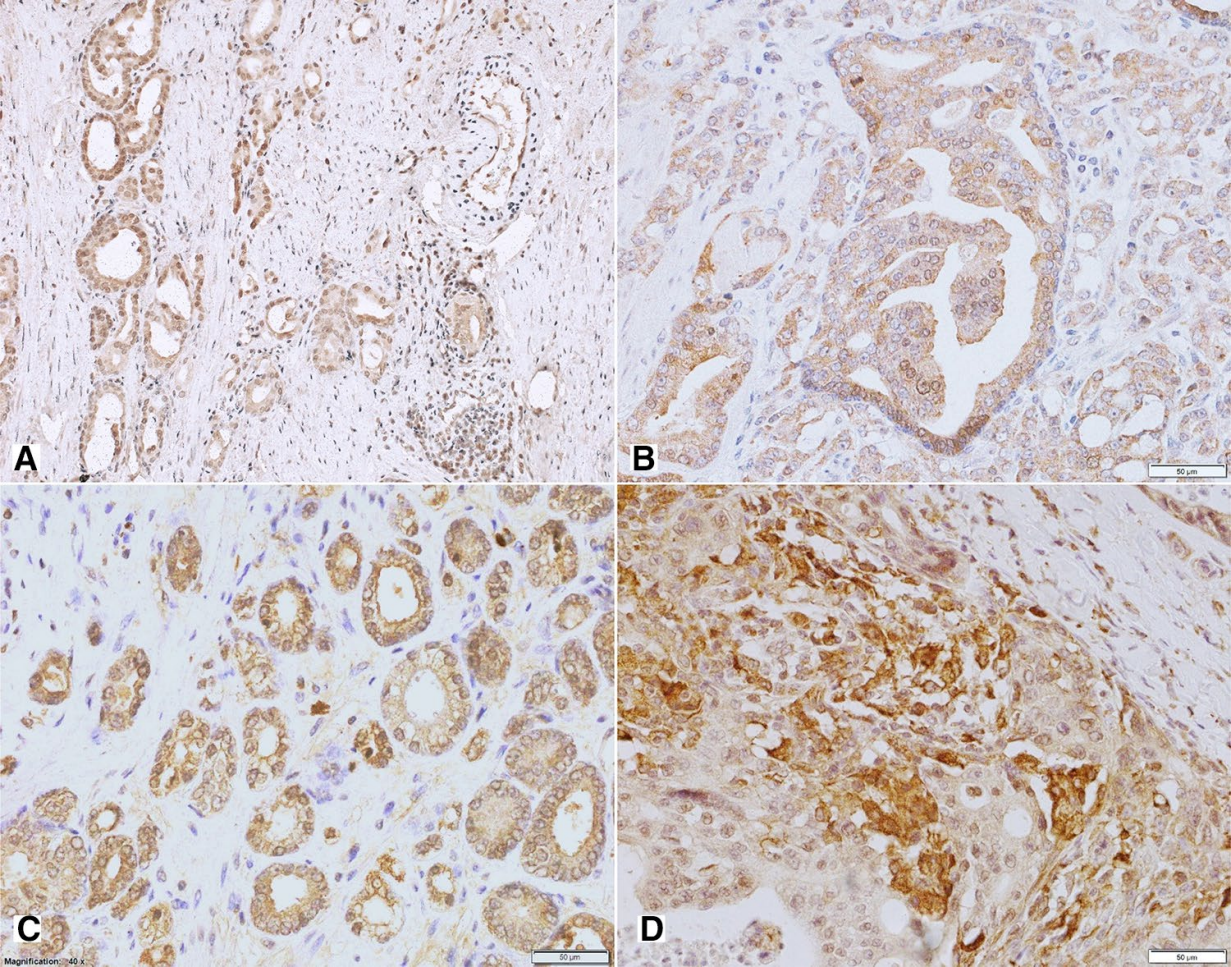
Tissue expression of Y5R. **A** High and moderate pattern expression in cancer glands and PIN, positive endothelia and lymphocytes (200×), **B** Y5R expression similar in infiltrating cancer glands and PIN (400×), **C **Y5R mixed pattern of strong expression in PCa (400×). **D** Immunoreactivity with diverse power, in part nuclear, in high-grade bone metastasis (400×)

In PCa, immunoreactivity was cytoplasmic and membranous showing mainly EI 2 (high EI in 76%). In 23/51 PCa cases and six bone metastases, Y5R staining was also nuclear. In PIN lesions, EI was high in 40% of cases (Figs. 1, 5A–C; Table 2). Y5R was also identified in cancer stroma elements such as the endothelial cells, scattered myofibroblasts and lymphocytes. Y5R expression in bone metastases was diverse, with EI 3 in 27% of cases (Figs. 1, 5D; Table 2).

### NPY system expression and PC progression

**Fig. 6:**
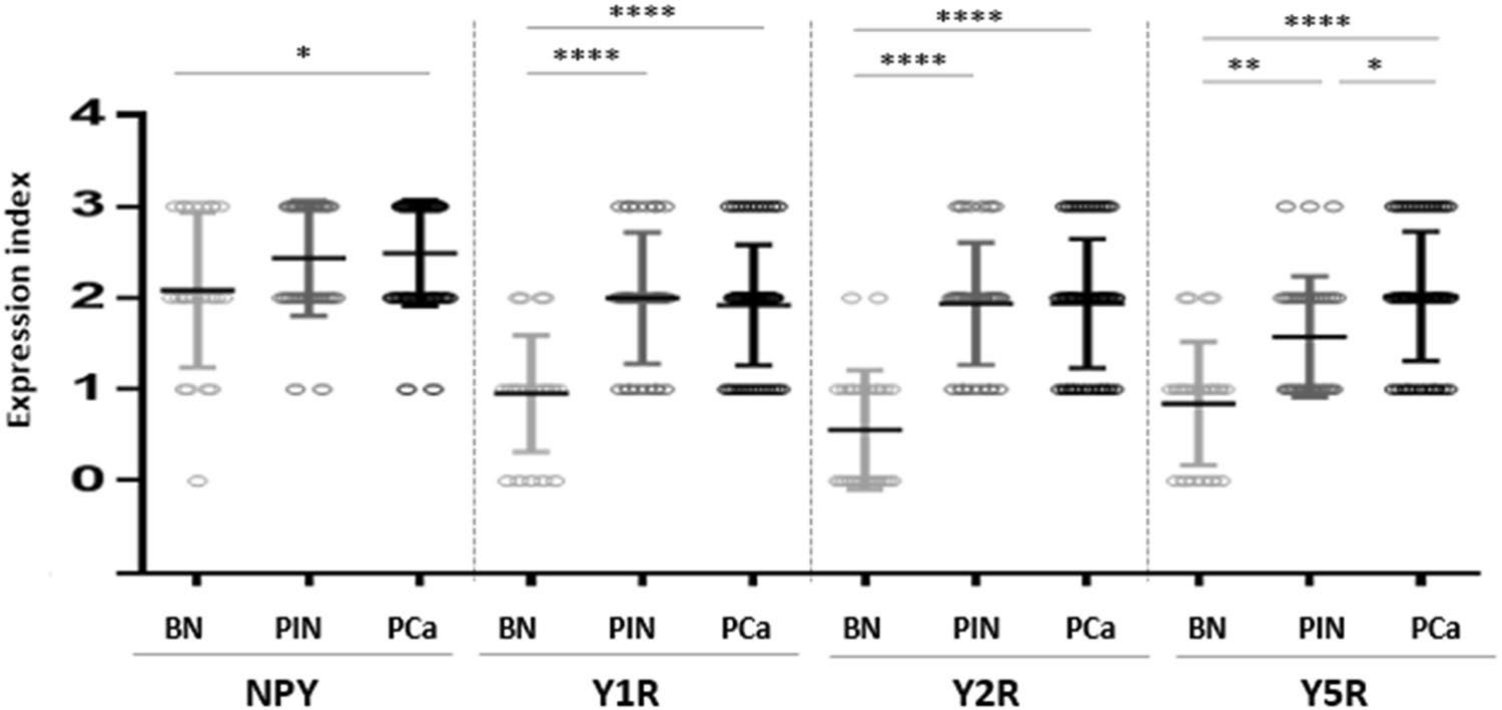
The comparison of the NPY system expression in PCa, PIN and BP. The expression index of NPY and Y1R, Y2R and Y5R was higher in PCa than BP (NPY *p* = 0.03; Y1R, Y2R, Y5R; *p* < 0.0001). In PIN, Y5R expression index was lower than that in PCa (*p* = 0.0002) Abbreviations: *BP* benign prostate, *PCa* prostate cancer, *PIN* prostate intraepithelial neoplasia

No significant differences between expression of NPY and its receptors in control BP cases and cancer-free prostate tissue from PCa cohort were found (NPY *p* = 0.60, Y1R, *p* = 0.86, Y2R *p* = 0.79, Y5R *p* = 0.26); therefore, all of them constituted the BP group. The increase in the NPY system expression started at early stages of PC. While the difference in NPY expression between BP and PIN did not reach statistical significance, NPY EI was significantly higher in PCa than BP (*p* = 0.03) and NPY expression in PIN did not differ significantly from invasive cancer (*p* > 0.99, Fig. 6). For all analysed NPY receptors, the significant increases in expression, as compared to BP, were observed for both PIN and PC (*p* < 0.01). Moreover, no statistically significant differences in the expression of Y1R (*p* = 0.344) and Y2R (*p* > 0.999) were observed between PIN and PCa. Y5R was the only NPY receptor with expression increasing with disease progression, as EI was significantly higher in PCa, as compared to the corresponding PIN (*p* = 0.0002, Fig. 6). In Pca, a positive correlation was found between Y2R and Y5R (correlation V-Cramer = 0.552; *p* < 0.001).

**Fig. 7:**
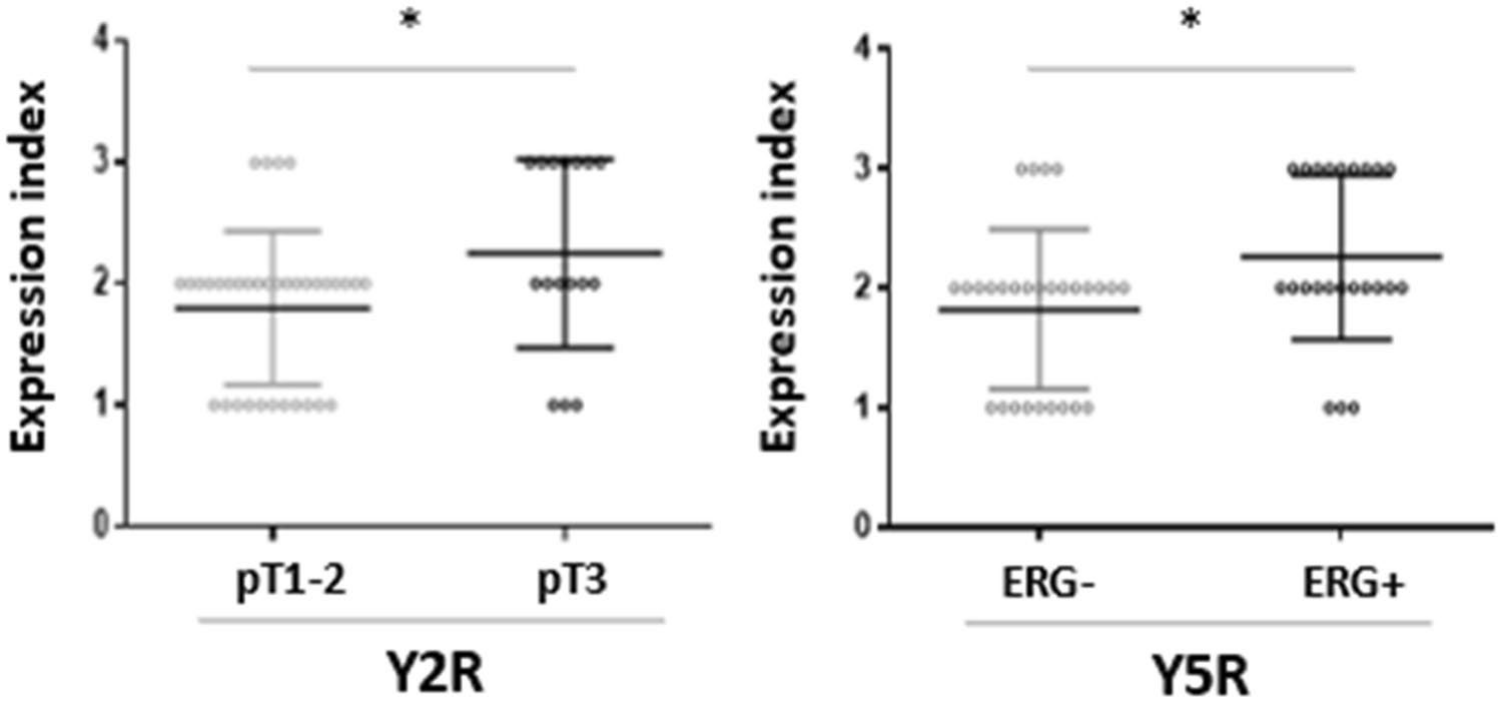
Association of the NPY receptor expression with clinicopathological features of PCa. **A** The comparison of Y2R expression index in pT1-2 and pT3 PCa. Y2R EI was higher in pT3 than pT1-2 PCa (*p* = 0.0272). **B** Y5R expression index (EI) in ERG-positive and -negative prostate cancer (PCa). Y5R EI is higher in ERG-positive than -negative PCa (*p* = 0.024). Abbreviations: *EI* expression index, *PCa* prostate cancer

No associations between the NPY system expression and pathoclinical features (patients’ age, tumour grade and proliferation index) were observed. Y2R was the only protein, which exhibited higher expression in pT3 PCa, as compared to tumours confined to the prostate gland (pT1-2) (*p* = 0.027) (Fig. 7A). Analysis of the NPY system associations with ERG expression revealed higher Y5R EI in ERG-positive PCa than in ERG-negative tumours (*p* = 0.024) (Fig. 7B). The other elements of the NPY system did not differ significantly between these two groups.

### Expression of NPY system is elevated in the invasive edge of PCa

**Fig. 8:**
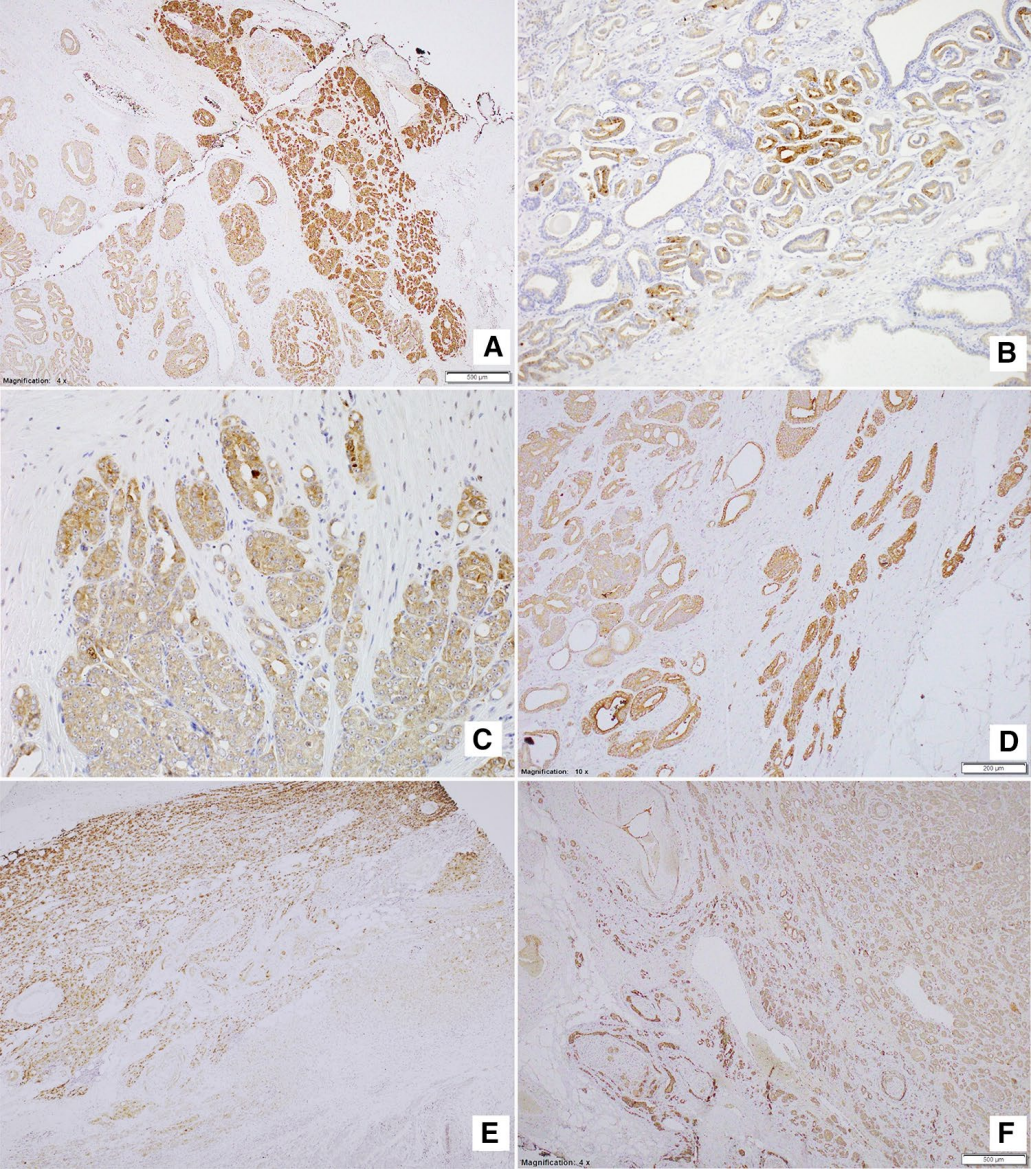
Enhancement in the NPY system expression on the invasive edge of PCa. **A** Increasing NPY staining gradient towards extraprostatic invasion (100×). **B** Heterogenous Y1R staining within the neoplastic infiltrate (200×). **C**, **D** Y2R staining intensification within the invasive front (400×, 200×). **E**, **F** Y5R staining gradient in the direction of EPE and PNI (40×, 100×)

**Fig. 9:**
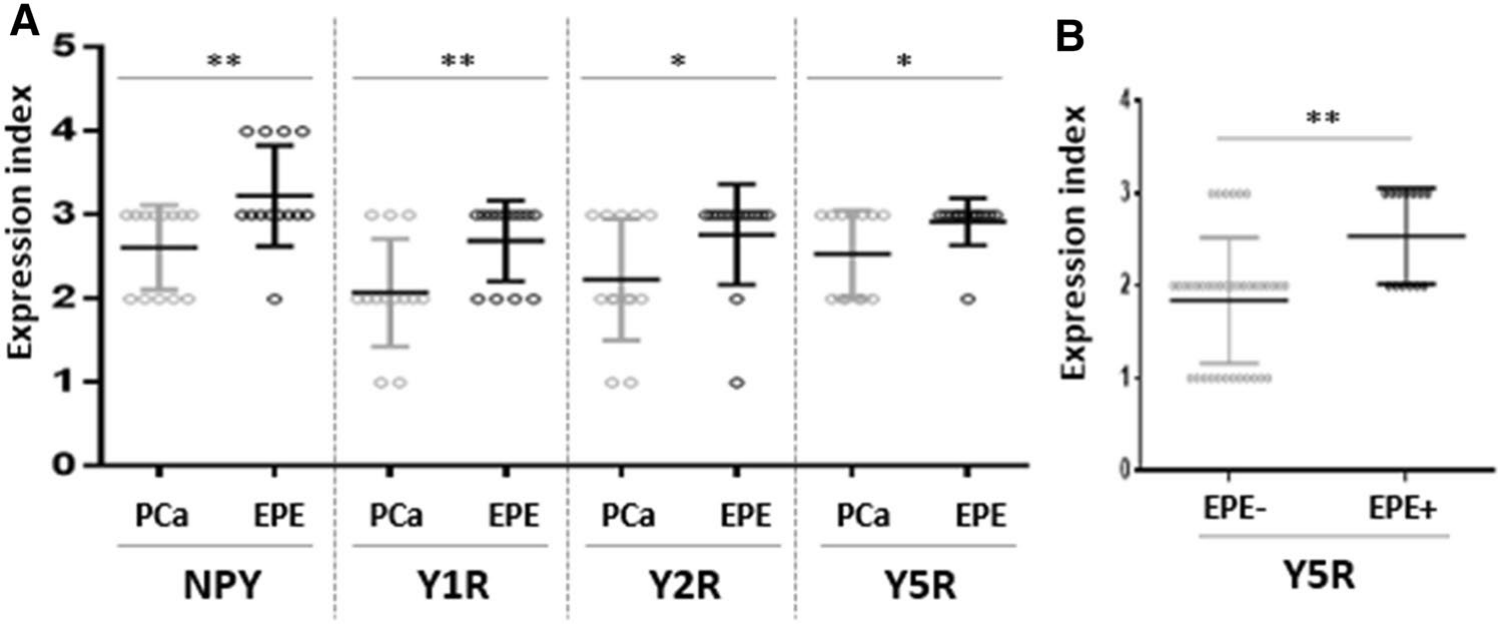
Neuropeptide Y (NPY) and NPY receptor system in prostate cancer (PCa) and extraprostatic extension (EPE). **A** NPY and Y1R, Y2R, Y5R expression index (EI) were higher in EPE areas than in the corresponding cancer infiltrated areas (NPY and Y1R, *p* = 0.008; Y2R, *p* = 0.0156, Y5R, *p* = 0.031). **B** Y5R in EPE-positive PCa was higher than that in EPE-negative tumours

The topographic analysis revealed a gradient of NPY system expression with immunoreactivity increasing towards the peripheral zone of cancer invasion. This characteristic spatial distribution was observed for NPY and all analysed receptors (Fig. 8). Consequently, the expression of all elements of the NPY system was higher in EPE areas, as compared to the corresponding main tumour mass (NPY and Y1R, *p* = 0.008; Y2R = 0.016; Y5R, *p* = 0.031; Fig. 9A). Moreover, when the cases in the study cohort were divided into those with or without evidence of EPE, the expression of Y5R in PCa was higher in EPE-positive cases (*p* = 0.002; Fig. 9B).

### PC cells with high expression of NPY and its receptors accumulate around nerves in areas of perineural invasion

**Fig. 10:**
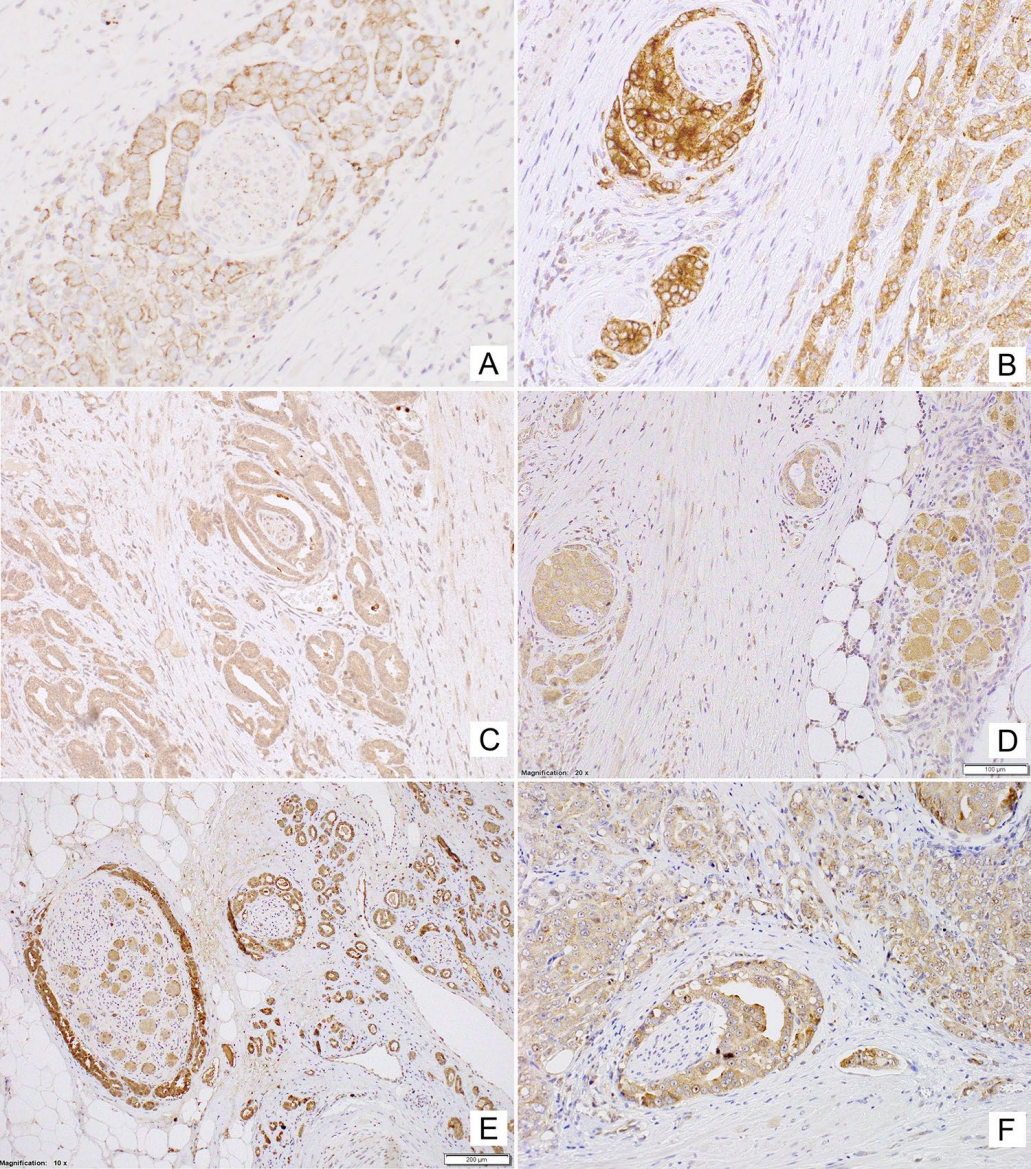
NPY system in cancer perineural invasion (PNI) areas. **A** NPY-positive nerve in PNI (100×). **B** Strongly enhanced NPY expression in PNI (200×). **C** Y1R immunoreactivity (200×). **D** Y1R expression within neoplastic cells in PNI equal to autonomic ganglia (200×). **E** Y5R expression higher in PNI than within ganglion cells (100×). **F** Increased Y2R expression within PNI and angioinvasion (200×)

**Fig. 11:**
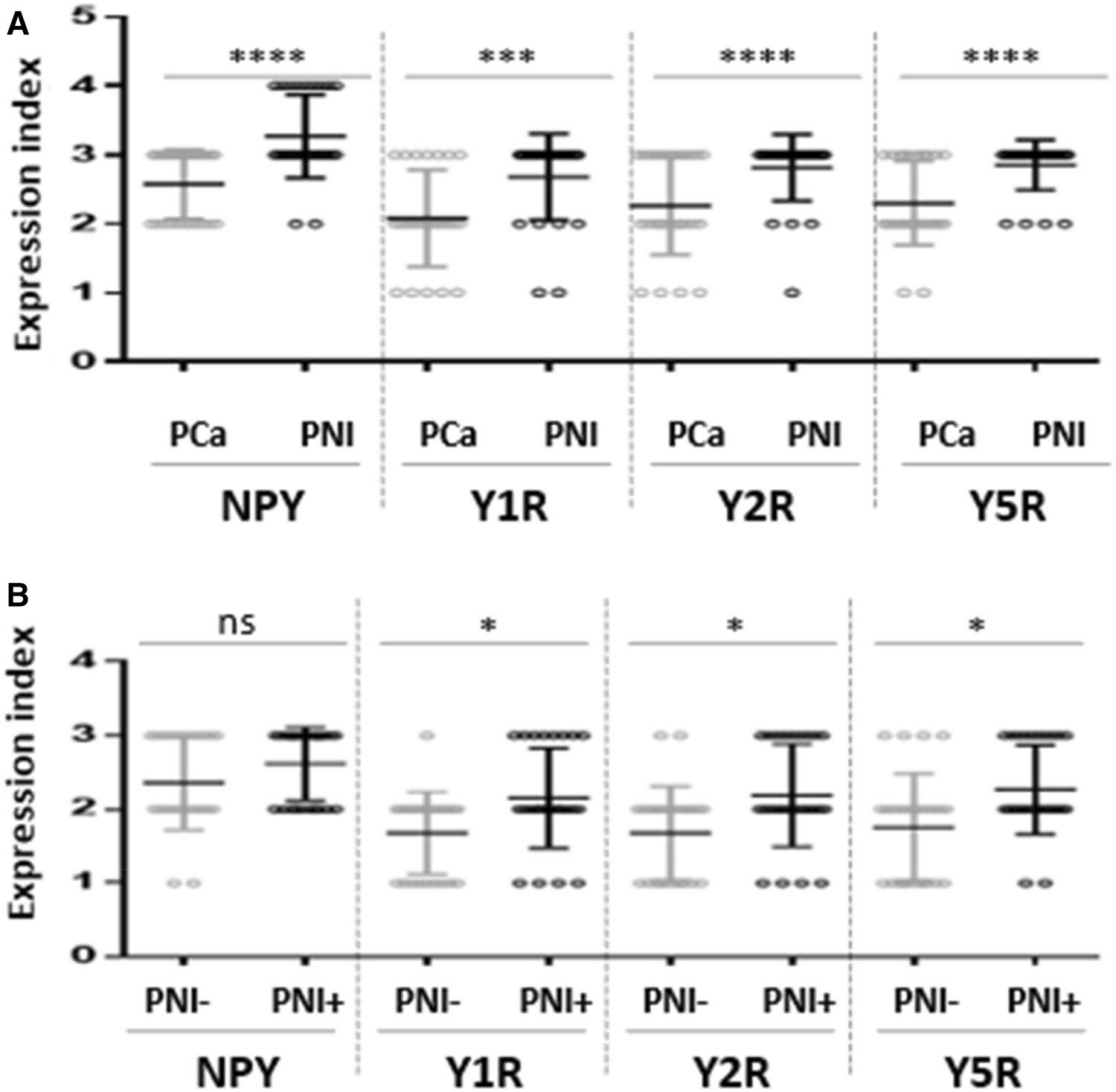
Comparison of the NPY system expression in PCa infiltration and PNI areas. **A** In PNI areas, EI was higher than in infiltration of corresponding PCa (NPY, Y2R, Y5R, *p* < 0.0001; Y1R, *p* = 0.0001). **B** The Y1R, Y2R and Y5R expression was higher in cases with PNI, as compared to those without PNI (Y1R, *p* = 0.015; Y2R, *p* = 0.012; Y5R, *p* = 0.015). Abbreviations: *EI* expression index, *NPY* neuropeptide Y, *PNI* perineural invasion

In addition to the overall increase in NPY system expression in the cancer invasion areas, an enhanced immunoreactivity was observed around nerves and ganglia (Fig. 10). In perineural areas of cancer infiltration, NPY EI was higher than in other tumour areas (*p* < 0.0001; Fig. 11A). Similarly, Y1R, Y2R and Y5R expression was higher in PNI areas than in the corresponding cancer (Y1R, *p* = 0.0001; Y2R, Y5R, *p* < 0.0001; Fig. 11A). While looking at the entire study group, Y1R, Y2R and Y5R expression was higher in cases with PNI, as compared to those without PNI (Y1R, *p* = 0.015; Y2R, *p* = 0.012; Y5R, *p* = 0.015; Fig. 11B). In contrast, the expression of NPY in PCa cases with and without PNI did not differ significantly (*p* = 0.09).

### Chemotactic effects of NPY

**Fig. 12:**
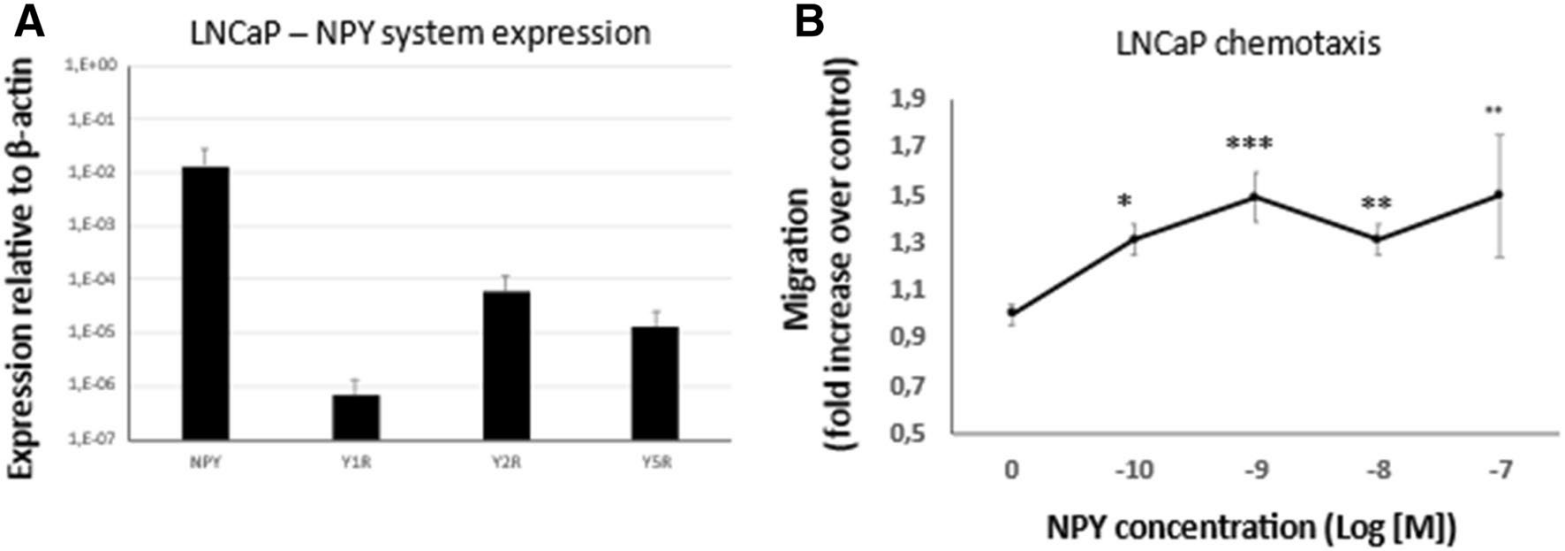
Chemotactic effects of NPY. **A** The expression of NPY and its receptors in LNCaP cells. **B** NPY acts as chemoattractant for LNCaP cells (*p* < 0.05)

The increased expression of NPY receptors in PCa cells surrounding nerves and ganglia suggested a role for the NPY system in perineural invasion. Hence, we sought to determine if neuronal NPY can act as a chemoattractant for PCa. To this end, we used androgen-dependent LNCaP cells, which express all three NPY receptors (Fig. 12A) in a transwell migration assay with the NPY gradient. NPY acted as a chemotactic factor for LNCaP cells and stimulated their migration at concentrations ranging from 10^–10^ to 10^−7^ M (Fig. 12B).

## Discussion

NPY and its receptors constitute a complex network involved in physiological processes, modulating cell proliferation, metabolism, angiogenesis, cellular stress and motility. NPY is a major co-transmitter in the sympathoadrenal system, released by nerve fibres that connect the cancer cell population with the neuroimmune–vascular environment by modulation of nutritional and inflammatory milieu (Zukowska-Grojec et al. 1998; Hansel et al. 2001; Tilan et al. 2013, 2015; Tan et al. 2017; Zhang et al. 2021). The multifaceted role of the NPY system in tumours and its differential function are driven by the variability in the receptor signalling pathways and peptide expression (Tilan and Kitlinska 2016). The presence of NPY and its receptors was described in several neoplasms, such as PCa, breast cancer, melanoma, hepatocellular carcinoma (HCC), cholangiocarcinoma, neuroblastoma, Ewing sarcoma and pituitary adenoma (Reubi et al. 2001; Kitlinska et al. 2005; Levy et al. 2006; DeMorrow et al. 2011; Hong et al. 2015; Lv et al. 2016; Pérez Tato et al. 2017). However, tissue expression of NPY receptor network has not been investigated in detail. Thus, this project aimed at performing a comprehensive immunohistochemical analysis of the NPY system tissue expression in primary PCa tumours, bone metastases, PIN and non-neoplastic prostate. In the second part of our research, we evaluated the chemotactic properties of the peptide in vitro*.*

Our study revealed high NPY expression in 96% of primary PCa, with a significant increase as compared to the corresponding BP. These findings are in agreement with previous reports (Rasiah et al. 2006; Ueda et al. 2013; Sigorski et al. 2021). The increased immunoreactivity was also observed in PIN. Similarly to the invasive cancer, in PIN, the high EI of NPY was detected in 93% of cases. In line with this, we did not find statistically significant differences in NPY expression between PCa and PIN. The high NPY levels in PIN have been demonstrated previously, with a higher percentage of NPY-positive cells in high-grade PIN than in cancer (Rasiah et al. 2006). NPY expression in transcriptomic analysis has also been shown to be higher in localized tumours, as compared to metastatic PCa (Alshalalfa et al. 2019). Altogether, our findings support the concept of NPY system upregulation as an early event in prostatic carcinogenesis (Rasiah et al. 2006; Haffner and Barbieri 2016). Interestingly, statistical analysis did not reveal any correlations between NPY and clinicopathological features, such as age, tumour grade and proliferation index. Similarly, most of other studies did not show such associations (Rasiah et al. 2006; Sigorski et al. 2021). The important finding in our study is the topographic relationship of the intensity of expression. The expression of NPY exhibited gradient intensification in the direction of the invasive tumour front, particularly towards EPE and PNI. In line with these observations, NPY EI was significantly higher in EPE, as compared to the main tumour mass. Furthermore, NPY expression at the invasive cancer front was equal or higher than within ganglion cells. The increased NPY EI was also observed in PNI areas and overall expression levels were higher in cases with PNI, as compared to PNI-negative cancers. The possible causes of higher NPY expression include increased peptide production in cancer cells under the influence of external factors, e.g. nerve growth factor (NGF) from the nerves, or internal factors caused by transcriptional upregulation, as well as changes in methylation of the coding gene. Another possibility is an uptake of NPY released from neighboring neural structures. Secretion of NPY from PCa cells is activated by noradrenaline which activates β2 adrenergic receptors and drives tumour progression (Khor and Baldock 2012; Dwivedi et al. 2021). Analogously, NPY-positive neurons in astrocytomas may serve as an additional source of different neuropeptides for neoplastic cells (Przedborski et al. 1988). Moreover, endothelial cells can capture NPY from the environment creating a paracrine proangiogenic loop (Zukowska-Grojec et al. 1998). PNI supports cancer cell survival by activating cellular mechanisms, such as antiapoptotic pathways (Ayala et al. 2006). Neuropeptides released from nerves initiate nerve–tumour cross talk and can induce neuritogenesis (Scanlon et al. 2015). Our previous study indicates a tendency towards lower PGP 9.5+ nerve density in the peripheral area of PCa in tumours with a high NPY expression (Sigorski et al. 2021).

In addition to the strong NPY immunoreactivity in PNI areas, PCa cells surrounding nerves and ganglia exhibited elevated expression of all NPY receptors. Several studies indicated a pro-migratory and chemotactic effects of NPY in a variety of normal and neoplastic cells (Medeiros et al. 2012; Tilan and Kitlinska 2016). Hence, such an accumulation of NPY receptor-positive tumour cells in areas of PNI suggests that NPY secreted from the neuronal cells may act as a chemoattractant for PCa. Here, we show that the exogenous NPY exerts chemotactic effect in PCa cells, as the androgen-sensitive LNCaP cell line migrated towards higher concentrations of the peptide. These observations implicate the role for the neuronal NPY in facilitating PNI in PCa. However, further studies are needed to identify NPY receptors responsible for these effects and design potential therapeutic strategies preventing this process.

NPY immunoreactivity presented with various staining patterns, from membranous and polarized luminal in benign prostate cells to the cytoplasmic and membraneous in PIN and cancer. The pre-existing normal prostatic glands that were interspersed within cancer infiltration showed a benign expression pattern. The membranous localization of peptide in normal luminal cells is associated with its secretion to the prostatic fluid. During carcinogenesis, neoplastic cells lose polarity, and glands lack a basal cell layer, which can be partly responsible for altered NPY staining pattern and profile of pro-cancerogenic activity. NPY was identified as an important factor responsible for promoting carcinogenesis by enhancing survival, proliferation and metabolic switch ( Tilan and Kitlinska 2016).

The NPY activities are mediated by the system of its G-protein-coupled receptors. All NPY receptors presented with significantly higher immunoreactivity in neoplastic glands than BP. The Y1R, Y2R and Y5R had cytoplasmatic–membranous staining. In case of Y5R, nuclear immunopositivity was also observed in 23 of 51 PCa and 6 of 11 bone metastases. Enhanced expression of receptors may result in the activation of MAPK and PKC pathways (Pellieux et al. 2000). Expression of the full spectrum of NPY receptors in cancer cells supports peptide multifaceted effects. The intracellular trafficking of receptors, including internalization after ligand binding, may also be one of the additional mechanisms of NPY accumulation in the cytoplasm (Babilon et al. 2013). Moreover, Y1R and Y5R heterodimerize, while Y2R and Y5R interact with each other indirectly, without evidence of dimerization. Both of these processes further modify the NPY function and biological effects (Walther et al. 2011; Czarnecka et al. 2019; Czarnecka and Kitlinska 2016). The neoplastic structures presented with higher expression of NPY receptors than the neighboring normal glands interspersed within the tumours. Moreover, Y2R expression was elevated in pT3, as compared to pT2 tumours. In PNI and EPE, the special areas of interest, EIs for all receptors were higher than in the main tumour mass. Furthermore, in the whole study group, the Y5R was higher in EPE-positive than in EPE-negative cases.

The role of NPY system and its receptors was studied most extensively in pediatric neoplasms—Ewing sarcoma and neuroblastoma. Overexpression of NPY in Ewing sarcoma and NB creates auto- and paracrine loops cooperating with endothelial cells, with pro-survival, pro-invasive and angiogenetic activities (Tilan and Kitlinska 2016). Furthermore, an increased level of NPY in the serum was a predictor of poor prognosis and disseminated disease (Tilan et al. 2015; Galli et al. 2016). Both Y2R and Y5R are proangiogenic receptors, which are selectively activated by NPY 3-36 form of peptide created by dipeptidyl peptidase IV-mediated cleavage (Lu et al. 2011; Tilan et al. 2015). In our analysis, we observed a strong NPY and Y2R immunoreactivity in angioinvasive cancer cells. The endothelium serves as a reservoir of NPY, which can act as a chemotactic factor for neoplastic cells. Hence, the endothelial NPY can potentially promote cancer cell intravasation. In neuroblastoma, increased Y5R expression is observed in tumour cells that infiltrate vascular walls (Galli et al. 2016). Y5R overexpression is induced via BDNF/TrkB pathway and increases neoplastic cell survival (Czarnecka et al. 2015). It is suggested that in the PNI area, NPY promotes angiogenesis. A similar mechanism was described in pheochromocytoma. Additionally, in normal breast tissue and benign breast lesions, Y2R was the dominant NPY receptor, while Y1R was the most abundant in cancer (Amlal et al. 2006). Activation of Y5R increases breast cancer cell chemotaxis towards NPY, proliferation and motility (Medeiros et al. 2012). On the other hand, in cholangiocarcinoma, NPY diminished proliferation and cellular motility via Y2R (DeMorrow et al. 2011). Recently it has been shown that in HCC, NPY is secreted by peritumoural hepatocytes and cross talks with overexpressed Y5R in tumour cells. Fibrosis and inflammation are mediated by TGFβ-induced hepatic NPY expression. In addition, enhanced peritumoural NPY drives chemotactic invasion of HCC cells. In HCC, Y1R and Y2R were downregulated, but Y5R was overexpressed at the invasive front of the cancer (Dietrich et al. 2020).

Bone as a metastatic site creates a special microenvironment for tumour growth. PCa preferentially metastasizes to axial bones, creating mainly osteoblastic lesions based on radiological assessment. The process of metastasis development is divided into tumoural escape and dissemination, adhesion, invasion and metastasis formation. Each of the processes is regulated by different factors and mechanisms of action (Wang et al. 2020). Complex interactions within the microenvironment of bone creates a reciprocal stimulating loop between PCa and osteoblasts. PCa cells secrete several trophic factors which activate osteoblasts and promote bone formation. The bone cells secrete chemotactic substances for cancer activation. Tumoural cells, by induction of osteoblast-like phenotype (osteomimicry), adapt to the bone microenvironment and increase its chance for survival (Rucci and Teti 2018). The process of metastasis remains under control of the nervous system and neurotransmitters (Maryanovich et al. 2018). The sympathetic and parasympathetic nervous systems control the NPY synthesis by osteocytes. PNI in PCa increases the risk for bone metastases 11-fold (Ciftci et al. 2015). The NPY system has an important role in bone metabolism, as it regulates osteoblasts and osteoclasts functions. However, its role in this process is complex and not fully understood (Chen and Zhang 2022). NPY receptors are present within bone cells and microenvironment and regulate bone mass and osteoblastic differentiation. In our study, bone metastases showed high NPY EI in 82% of cases. However, other studies indicated lower NPY expression in metastasis and in metastatic tumours with neuroendocrine differentiation (Alshalalfa et al. 2019). Analysis of the receptor system in bone metastases showed high Y1R and Y5R expression (64%), in contrast to Y2R, which was high only in 36% of cases. Statistical analysis revealed a positive correlation between Y2R and Y5R expression in the PCa group. Studies focusing on Ewing sarcoma revealed that tumour cells with high NPY expression metastasize preferentially to the bone, while the activity of the NPY/Y5R axis induced by hypoxia is crucial for their osseous dissemination (Hong et al. 2015; Lu et al. 2022). However, the additional role for NPY in the formation of premetastatic niche within the bone cannot be excluded. NPY partially controls the bone homeostasis. Activation of Y1R inhibits osteoblasts activity and, therefore, bone formation. PCa, which infiltrates bone, may keep and store peptides leading to loss of osteoblasts inhibition. In addition, NPY modulates cellular communication among other microenvironmental cells in prostate stroma, such as cancer-associated fibroblasts, immune cells and fibroblasts; with nerve fibres being the main source of NPY (Geloso et al. 2015). In our study, we observed Y1R expression in PCa stroma fibroblasts, but there is no data about the role of Y1R in fibroblasts. Cancer-associated fibroblasts release growth factors and chemokines, and regulate tumour growth and immune resistance. Y1R mediates pro- and anti-inflammatory responses, which are part of the immunomodulatory properties of the NPY system (Chen et al. 2020)**.**

NPY has also been studied as a potential additional prognostic factor in localized and metastatic PCa, with conflicting results. A combination of systemic NPY and PSA levels showed 81.5% sensitivity and 82.2% specificity for PCa diagnosis (Ueda et al. 2013). Low NPY gene expression was associated with adverse genomic features and high-risk PCa according to D′Amico's definition*, *which is composed of PSA, tumour grade and stage status (D’Amico et al. 1998). On the other hand, in multivariate analysis, patients with higher NPY immunostaining had a higher risk of PSA relapse after prostatectomy than those with low NPY patients (Rasiah et al. 2006), while another study reported increased mortality among PCa patients with high pro-NPY (Iglesias-Gato et al. 2016)*. *Our study did not find any significant correlations between the NPY system and tumour stage, grade, patient age and proliferation index. Proliferative index Ki-67 in our analysis was 4% and was comparable with other studies (Berlin et al. 2017). Frequency of ERG + PCa in our work was 50%, in concordance with current literature (Kaczmarczyk et al. 2013; Sigorski et al. 2021). ERG status could be an unfavorable prognostic factor in PCa. Some authors described that ERG-positive PCa cases have higher NPY expression than ERG-negative tumours. In our cohort, we found only a correlation between high Y5R expression and ERG-positive status. Alshalafa et al. revealed association of low NPY expression and ERG-positive subtype (5% of total PCa) with the highest risk of developing metastasis (Alshalalfa et al. 2019). Another group showed a positive correlation between high pro-NPY and ERG-positivity; however, without any associations with clinical outcomes (Kristensen et al. 2018). Proteomic profiling study of primary PCa revealed that high pro-NPY expression, regardless of the ERG status, was associated with an increased PCa-specific risk of death, especially in patients with Gleason score ≤ 7 tumours. This association with a decreased survival was independent of the Gleason score (Iglesias-Gato et al. 2016). Hence, further studies are required to elucidate the prognostic value of the NPY system expression, with clear distinction between the clinical significance of the circulating levels of the peptide and tissue expression of the NPY and its receptors.

## Conclusions

The evidence for the role of NPY in PCa development and progression is growing. Previous reports suggested its multifaceted effects ranging from regulation of proliferation, chemoresistance to metabolic reprogramming (Hansel et al. 2001; Körner and Reubi 2007; Ding et al. 2021). Our data clearly indicate an association of the elevated NPY system expression with invasive properties of PCa and its perineural spread. However, functional studies are necessary to support the above findings and provide direct experimental evidence for the involvement of the NPY and its receptors in PCa progression to the invasive and metastatic phenotype. Altogether, previous studies and our current findings warrant further investigations into the role of NPY in PCa biology and its potential implications for the disease diagnosis, stratification and therapy (Yi et al. 2018; Maschauer et al. 2019; Chastel et al. 2020; Worm et al. 2020; Hoppenz et al. 2020; Ding et al. 2021).

## Data Availability

The datasets generated during and/or analysed during the current study are available from the corresponding author on reasonable request.
